# Design, assembly, alignment and application of a versatile, open-source, single-pixel microscope

**DOI:** 10.1038/s41598-025-03125-1

**Published:** 2025-05-22

**Authors:** Samuel I. Zapata-Valencia, Heberley Tobón-Maya, Luis Ordoñez, Andrea Farina, Jesús Lancis, Enrique Tajahuerce

**Affiliations:** 1 Institute of New Imaging Technologies (INIT), Universitat Jaume I, Castelló de la Plana, 12071 Spain; 2 Dipartimento di Fisica, Politecnico di Milano, Piazza L. da Vinci 32, Milan, 20133 Italy

**Keywords:** Single-pixel imaging, Computational imaging, Microscopy, Open-source design, Imaging techniques, Microscopy, Imaging and sensing

## Abstract

Single-pixel microscopy (SPM) is an emerging imaging technique in which a sample is illuminated with a series of micro-structured light patterns, typically generated by a digital micromirror device (DMD). After interaction with the sample, light is collected by a bucket detector, and the image is reconstructed through computational algorithms, such as basis transformations or compressive sensing. DMD achievable framerates and wide spectral range has allowed SPM to develop a wide range of applications, including polarization state analysis, phase imaging, and fluorescence lifetime measurements. To achieve optimal performance in these applications, a precise system configuration is required ensuring the effective projection of the structured light patterns. Nevertheless, the incorporation of a DMD introduces additional complexity, particularly in alignment, which can significantly affect system performance if not properly addressed. This work presents a comprehensive framework for the design, assembly, and alignment of a modular, open-source SPM system. The proposed procedures minimize aberrations introduced during construction and ensures the accurate projection of structured light patterns onto the sample. The modular design facilitates integration across multiple illumination sources and enables simultaneous brightfield transmission and reflection imaging. The proposed system achieves resolution near the diffraction limit, surpassing previous SPM configurations without requiring numerical or optical enhancement techniques. Performance validation through imaging experiments on both biological and non-biological samples demonstrates the system’s robustness and versatility. By providing detailed design and assembly instructions, this work contributes to the openness and reproducibility of SPM and serves as a valuable resource for researchers aiming to build high-performance, customizable single-pixel imaging systems.

## Introduction

Array detectors are traditionally integrated into imaging systems to enable digital acquisition and digital processing, which facilitate the visualization of samples. However, these capabilities depend on the detector specifications, which impose some limitations, especially for imaging outside the visible region of the electromagnetic spectrum^1,2^. In this way, single-pixel imaging (SPI) has emerged as a promising unconventional imaging technique, taking advantage of the simplicity and variety of market-available bucket detectors. In SPI, the array detector is replaced by a single-pixel detector together with a spatial light modulator (SLM)^3^. Conventional single-shot imaging becomes now into a sequential acquisition of intensity values provided by the bucket detector, while the SLM modulates the sample’s information. These changes shift the image retrieval process to computational efforts, reducing the complexity of the detectors while increasing their versatility in acquisition capabilities^4–9^.

In principle, any SLM can be utilized to modulate the sample information in SPI. However, the faster the modulation the less time spent on the intensity acquisition. According with that, the digital micromirror device (DMD) has emerged as an essential SLM for SPI because it is capable of providing binary modulation of light up to 32 kHz^10^. This device modulates the light by tilting micro-sized mirrors to two positions at high-speed rates^11,12^. Nevertheless, the tilt angle makes the DMD alignment a rather labor-intensive task. To simplify the alignment, total internal reflection prisms (TIR) can be incorporated in some applications^13^. However, the use of TIR restricts the system to the specific design wavelength range, which is commonly in the visible spectral range, limiting the flexibility of the device for wider spectral bands and nonlinear applications^14^.

The combination of such fast devices with novelty compressive sampling techniques has profiled SPI as a promising field of study and technology development^10,15,16^. In particular, the development of single-pixel microscopy (SPM) techniques has made it possible the use of specialized detectors suitable for spectral ranges and applications where the accessibility of array detectors decreases due to high cost^2^ or unavailability^9,17,18^. Specifically, this has enabled the integration a variety of light-detection frameworks, such as highly sensitive detectors^13^, high temporal-bandwidth sensors^4,19,20^, and wide-spectral light sensors ranging from SWIR to the X-Ray spectrum^21,22^.

Despite the theoretical foundations for SPM are well established, practical studies about the setup, configuration and standardization for SPM design, alignment and testing remain scarce^23,24^. For new researchers, aligning the optical system, particularly due to the presence of the DMD, can be challenging as this is not well documented in technical reports^11,12^. Recently, some notable efforts have been done in this direction, in wavefront shaping applications^24^ and in SIM techniques.

To bridge this gap, this scientific report presents a reproducible framework for constructing and aligning a single-pixel microscope integrating both transmission and reflection modalities. This study also offers a comprehensive standardized design guide that systematically addresses the challenges of incorporating a DMD into SPM. It discusses the coupling of adaptive optical elements, the constraints on the technique resolution in SPM. These aspects have not been analyzed together in detail in the framework of SPM design and implementation before. As well, a high spatial resolution sensor to control the quality of the projected patterns, and the possible addition of data-fusion techniques has been integrated. Additionally, a dual setup is demonstrated, enabling the simultaneous acquisition of sample information in both bright-field transmission and reflection modes. We validate the design through imaging various biological and non-biological samples, showing the versatility and potential of this SPM configuration for diverse applications.

## Single-pixel microscopy (SPM) operational and design factors

In conventional imaging, the image of an object is typically captured in a single shot by using millions of optical sensors contained in a CMOS or CCD array. In contrast, in SPM the object is sampled through a series of light patterns, usually encoding functions of a specific basis, while the light intensity is recorded sequentially onto a single-pixel detector^3^. The image is then reconstructed by means of an inverse transformation. In the literature, two main optical configurations are described to achieve this, active SPM and passive SPM, also referred as structured illumination and structured detection, respectively.

**Fig. 1:**
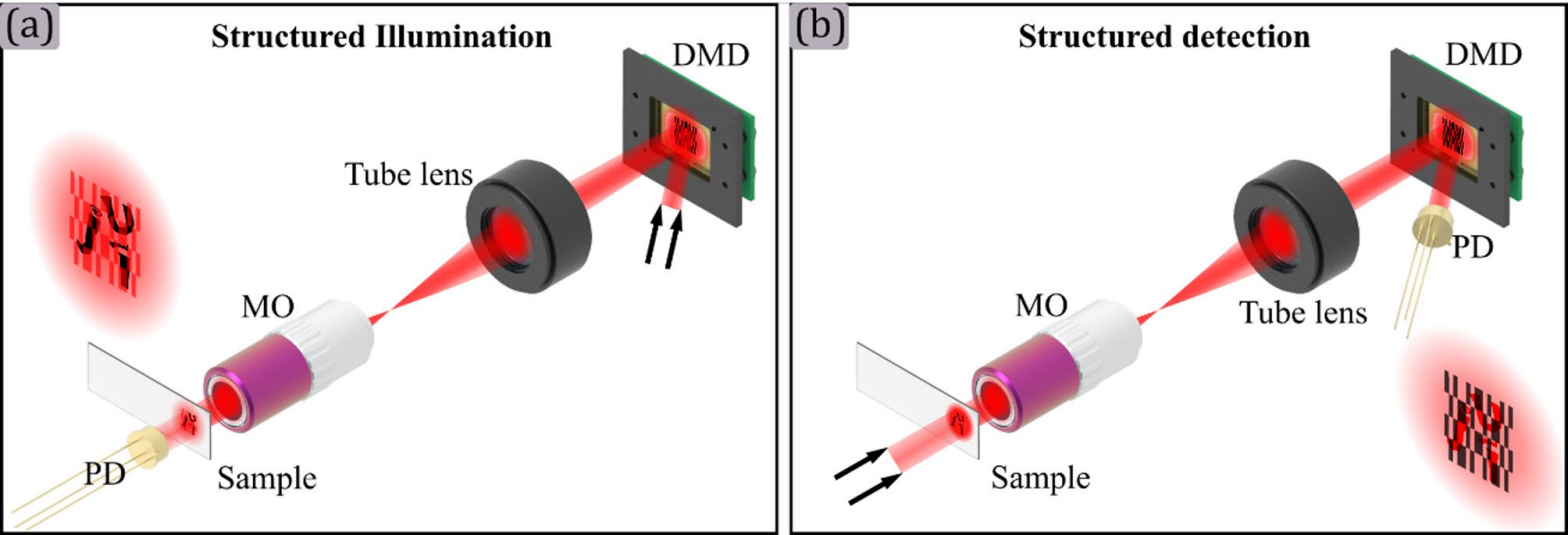
Single-pixel microscopy configurations. Panel (a) shows a structured illumination configuration where a plane wave illuminates the DMD, and the modulated light pattern is projected over the sample. Then, the scattered light is integrated by the photodiode (PD). In contrast, panel (b) shows a structured detection setup, where the sample is always illuminated, and its optical image is magnified and projected over the DMD, which modulates the reflected light towards the PD.

In active SPM, the set of patterns, encoded in the SLM, are demagnified by the microscope setup and projected on the sample plane. Then, after interacting with the sample, the light is collected using the bucket detector, as is shown in panel (a) of Fig. 1. This configuration minimizes sample degradation by illumination, due to the spatial structure of the light patterns, which reduces photobleaching in fluorescence applications. After the pattern projection, light can be collected in either reflection or transmission configurations, enabling the simultaneous acquisition of multimodal information about the sample. Despite these advantages, a critical challenge in active SPM is optimizing the balance between spatial resolution, and binning strategies, which impact the achievable image quality and remain underexplored in prior studies.

In contrast, in passive SPM a magnified image of the object is produced on the DMD plane. The light interacting with the DMD for each pattern is collected by the bucket detector, as is shown in panel (b) of Fig. 1. This approach enables the acquisition of modulated information and its complementary at the same time, taking advantage of the binary nature of the DMD^2^. If the DMD is replaced by a conventional camera, the setup converts seamlessly into a conventional imaging system.

In this work, the structured illumination approach is employed to design an SPI-based microscope that prioritizes sample preservation. This method not only minimizes photodamage but also facilitates the rapid integration of multiple light sources and detectors enabling versatile data acquisition strategies, both in transmission and reflection configurations. As will be further discussed, this flexibility allows simultaneous imaging across multiple detection channels, paving the way for multimodal analysis and enhancing the adaptability of the microscope to various experimental requirements.

### Hadamard basis, sampling and reconstruction

**Fig. 2:**
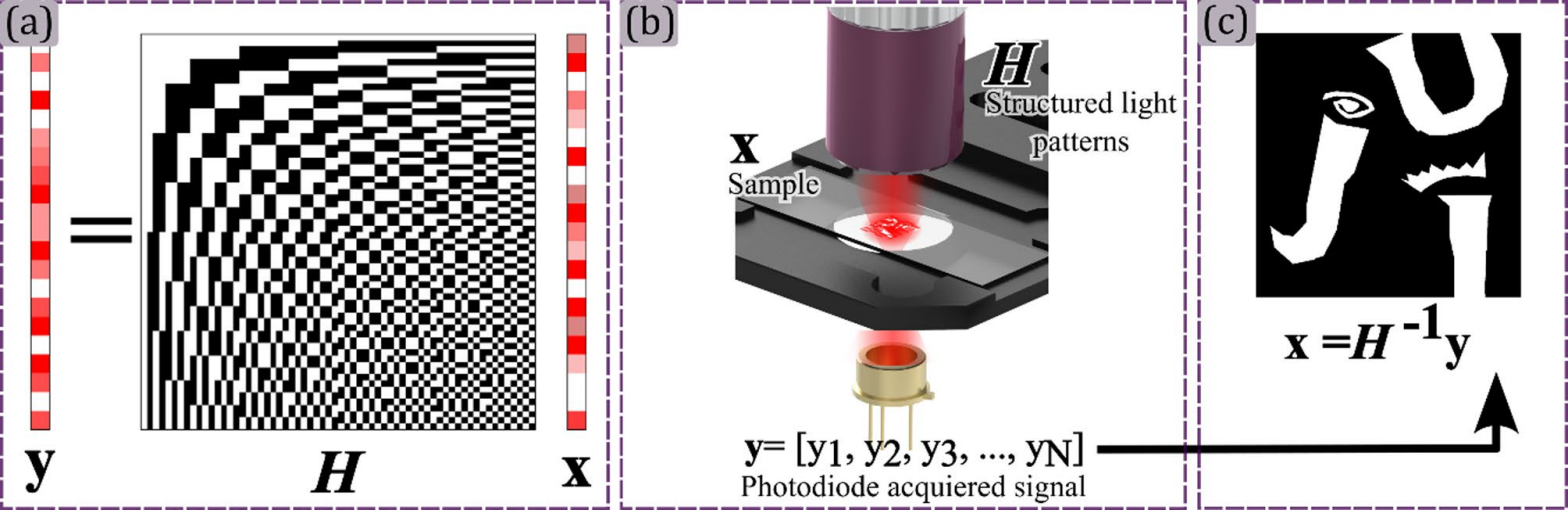
HSPM acquisition and image retrieval processes. In panel (a), the intensity vector $\:y$ as the product of the Hadamard basis $\:H$ matrix with the sample information vector $\:x$. In panel (b), a description of how each component of the intensity vector $\:y$ is acquired in the microscopic setup. Finally in panel (c), the inverse Hadamard transform $\:{H}^{-1}$ is computed over the intensity vector $\:y$ to retrieve the sample information.

Several function bases can be used to encode sample information in SPI^25–30^. However, the DMD’s binary modulation characteristic makes a binary bases particularly advantageous. Therefore, the Hadamard basis $\:H$ is used to generate binary Hadamard patterns, which are projected onto the by means of structured illumination. Having this in mid, in this scientific report the Hadamard basis will be used as the support for SPM. In a Hadamard-based single-pixel microscope (HSPM), each function of the Hadamard basis is projected over the sample as a two-dimensional rectangular light pattern. Each Hadamard function, containing ± 1 values, is projected by combining two complementary light patterns, one codifying the positive and another the negative component of the Hadamard function. The pointwise product between each Hadamard pattern $\:H$ and the sample $\:x$ is integrated by a bucket detector, creating an intensity vector $\:y$, that encodes the sample information, as is illustrated in Fig. 2 panels (a) and (b).

To reconstruct the object’s image $\:x$, the intensity vector $\:y$ is multiplied by the inverse Hadamard matrix $\:{H}^{-1}$. Given the symmetry property of the Hadamard basis, $\:{H}^{-1}=H$, this simplifies the reconstruction process to a straightforward matrix multiplication, as shown in Fig. 2 panel (c). This streamlined approach offers an efficient pathway to retrieve high-fidelity images from a minimal number of intensity measurements, a key advantage of Hadamard basis in SPI.

### Field of view and resolution

Once the operational principle of SPM has been clarified, is necessary to define the design parameters in terms of the pursued image quality. In this section the relations between the pixel pitch, the resolution and the numerical aperture (*NA*) in SPM are discussed. As well as in optical imaging, there are two parameters of paramount importance for assessing image quality in SPM: namely the field of view (FOV) and the spatial resolution. The FOV in Hadamard-based SPM is determined by the size of the demagnified projected pattern by the optical system. Equation (1) describes the relation for a square pattern of $\:n$ pixels along each axis; where $\:M$ is the magnification of the system, and $\:{p}_{x}$ is the size of the smallest pixel of the Hadamard pattern. It is interesting to note that the former variable is not necessarily the size of the SLM pixel, in our case, the size of the DMD micromirrors. In fact, it is possible to apply binning to have one pixel of the Hadamard pattern formed by more than one adjacent SLM pixel.

1 $$\:FOV={\left(\frac{{p}_{x}\:n}{M}\right)}^{2},$$

On the other hand, spatial resolution refers to the ability of an optical system to distinguish between two points or objects that are close together. It is typically defined as the smallest distance between two objects that can be clearly resolved or identified as separate entities rather than as a single blurred point. When all the Hadamard base is sampled and no compressive sensing strategies are applied, the smallest resolvable detail in SPM is given by the minimum between the geometrical demagnification of the smaller pixel of the projected Hadamard pattern,

2 $$\:{\Delta\:}{r}_{geo}=\frac{{p}_{x}}{M},$$

and the spatial resolution of the optical system limited by the well-known Rayleigh criteria,

3 $$\:{\Delta\:}{r}_{dif}=0.61\frac{\lambda\:}{\:NA},$$

where $\:\lambda\:$ is the illumination wavelength and $\:NA$ is the numerical aperture of the system. If $\:{{\Delta\:}r}_{geo}<{{\Delta\:}r}_{dif}$ the smaller patterns are not correctly projected over the sample plane due to a diffraction limitation of the system. If this is the case, the blurred patterns projected over the sample plane would compromise the retrieved image resolution. Then, it is convenient to apply binning by increasing the pattern pixel size. In this way, we optimize both FOV and resolution, avoiding unfavorable projection. To bring in context the reached resolution in the SPM state of the art in Table 1 the information presented by Wu et al.^31^ has been adapted. The performance of different SPM developments is shown, and the imaging conditions are detailed.

**Table 1:** List of the operational performance in single-pixel microscopy.

Previous reported	Imaging conditions	Resolution performance in terms of 1951 USAF target elements
^32^2013	NA	Group 4, element 5*
^2^2014	MO = 36x NA = 0.52	Group 7, element 3*
^25^2017	MO = 10x NA = 0.4	Group 7, element 3*
^13^2018	MO = 10x NA = 0.25	Group 7, element 4*
^31^2021	MO not applicable Magnification rate 0.1 Microscope with 300 mm and 30 mm focal length lenses	Group 6, element 6
^33^2023	MO = 10x NA = 0.25	Group 6, element 6
^16^2025	MO = 50x NA = 0.5	Group 9, element 1

*Computed based on the article presented results.

Considering the previous operational parameters for SPM, and the state-of-the-art performance, the design of a modular HSPM is presented in the following section. The proposed design is optimized over the operational parameters to reach high resolutions by minimizing the system aberrations and introducing adaptive optical elements. The design allows the incorporation of multiple illumination sources and different detectors. The assembly instructions, the system to correctly project the patterns over the sample plane, and the DMD alignment with the microscope setup are detailed. As well the construction of the proposed design and experimental validation will be provided. These considerations for the HSPM validate the proposed design as a reproducible framework for SPI in terms of hardware, assembly procedures and detector integrations.

## Single-Pixel microscope design

### Proposed system overview

**Fig. 3:**
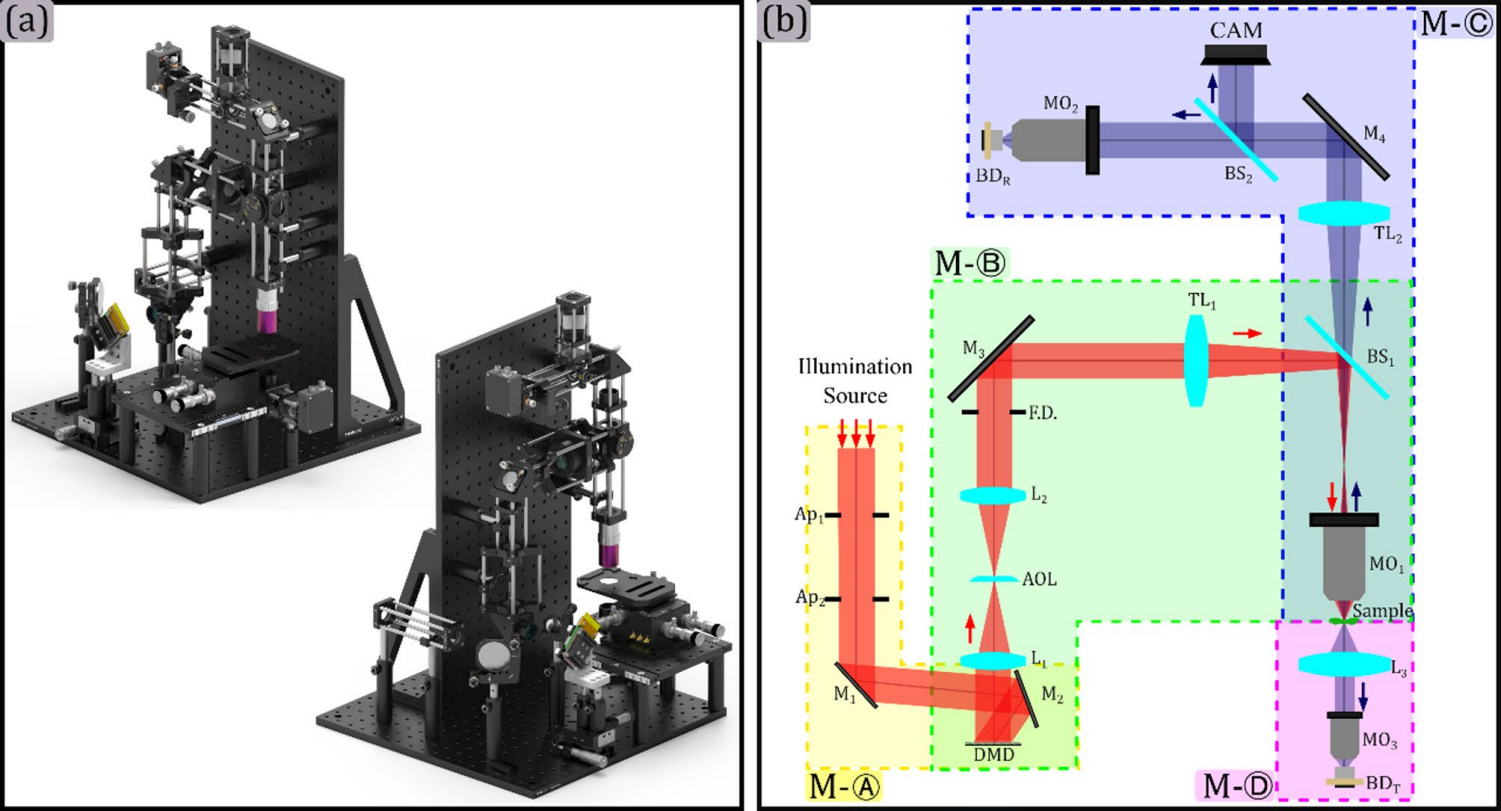
Proposed single-pixel microscope. In panel (a) a render in two perspectives is shown. In panel (b) a schematic and modular division is detailed. All the corresponding microscope modules are delimited by dotted lines, being M-Ⓐ the illuminating coupling module, M-Ⓑ the projection module, M-Ⓒ the reflected-light sensing module, and M-Ⓓ the transmitted-light sensing module. The detailed element in the schematics corresponds to Ap_**i**_: aperture ith, M_**i**_: mirror ith, L_**i**_: lens i^h^, FTL: focus tunable lens, FD: field diaphragm, TL_**i**_: tube lens ith, BS_**i**_: beam splitter ith, MO_**i**_: microscope objective ith, BD_**i**_: bucket detector ith.

The HSPM presented in this work is shown in Fig. 3. A full render perspective is shown in panel (a). A modular design allowing versatility and robustness was implemented. In panel (b) a detailed schematic of all modules is shown. The design consists of a DMD-based structured illumination system, a collection optics module, and a high-sensitivity single-pixel detector. A key concept of the proposed microscope is that, unlike most of the reported SPM^2,13,25,31,33,34^, the presented system is designed to operate in both reflection and transmission modes, allowing for multimodal imaging of biological samples. This significantly reduces the cost of optics, particularly for applications related to IR microscopy. In traditional dual-acquisition setups, a complete imaging system with costly IR optics must be constructed for transmission imaging. However, this setup addresses wavefront distortions and allows for real-time autofocus adjustments by utilizing adaptive optics elements.

A major challenge in SPM implementation is the integration of DMDs, tilt angle, and optical alignment, which significantly impact system performance. This section provides a systematic approach to addressing these issues, ensuring robust and reproducible system operation. In the proposed system, collimated illumination from the light source gets into the system through the coupling module M-Ⓐ; then, the light is redirected to the DMD and is reflected perpendicularly in the direction of the Projection module M-Ⓑ. The DMD, the field diaphragm, and the sample plane are conjugated in this module. The Projection module M-Ⓑ generates structured illumination by forming a demagnified image of the DMD patterns on the sample plane. Finally, the light reflected or transmitted by the sample is acquired by the Reflected-light sensing M-Ⓒ and Transmitted-light sensing M-Ⓓ modules, respectively, in a single-pixel dual detection configuration.

The role, design, and assembly instructions of each module in the microscope are explained in detail in the following subsections. The Helmholtz reciprocity theorem of light^35^ is used to align the microscope from the inside to the outside, guaranteeing the proper integration of the DMD into the microscopy system.

### Projection module

**Fig. 4:**
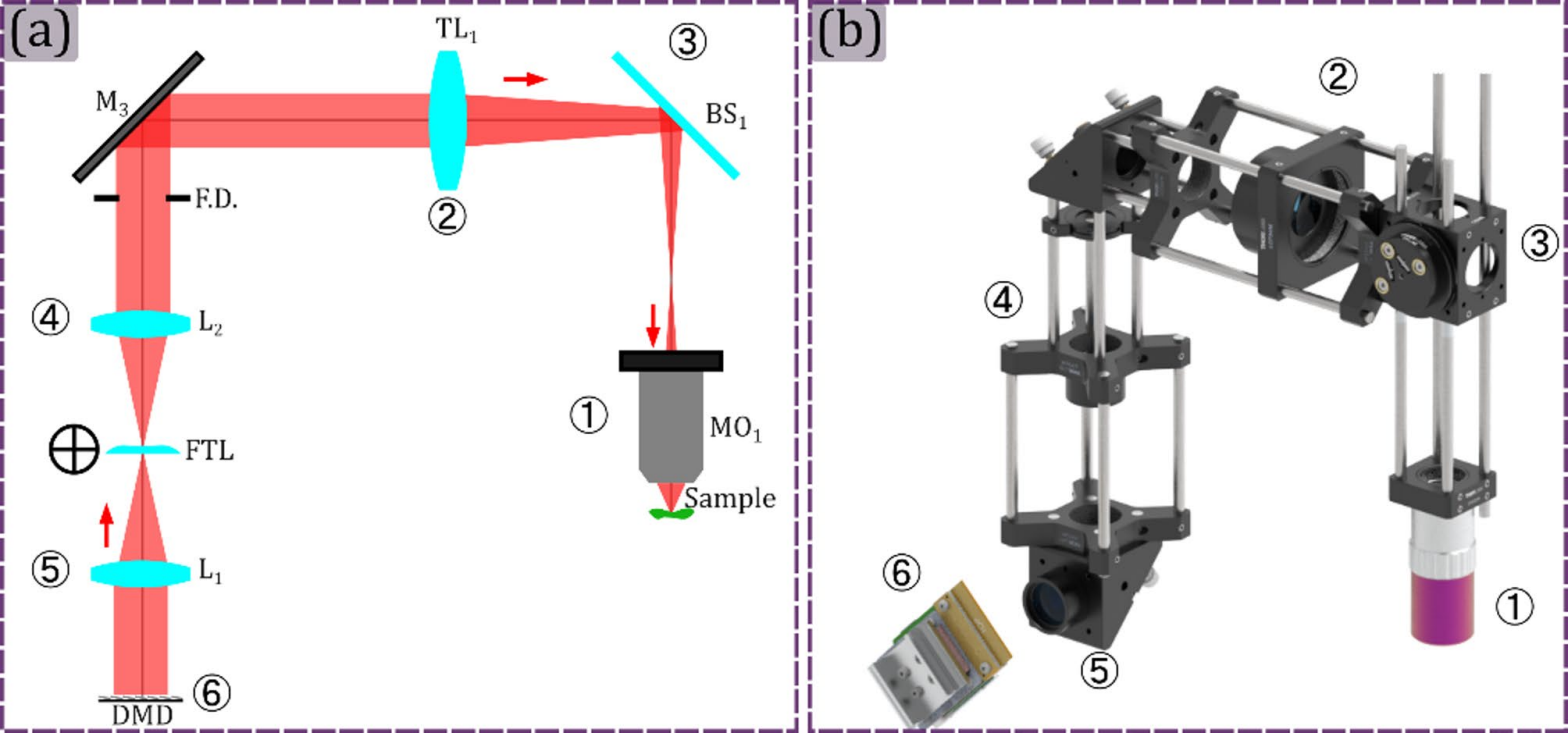
Projection module. In panel (a) the projection module schematic is shown. The optical elements are labeled both with their corresponding acronym and numerically. In panel (b) the rendered 3D view of the projection module is shown, where each optical element is referenced using the corresponding number from panel (a). The detailed element in the schematics corresponds to M_**i**_: mirror ith, L_**i**_: lens ith, FTL: focus tunable lens, FD: field diaphragm, TL_**i**_: tube lens ith, BS_**i**_: beam splitter i^th^, MO_**i**_: microscope objective ith.

The Projection module is set to correctly project the structured light patterns onto the sample plane. To do this, an image of the digital patterns codified in the DMD must be formed in the sample plane. In this module, two lenses of equal focal length are located in a 4f configuration after the DMD, followed by a tube lens and a microscope objective, as is shown in panel (a) of Fig. 4. The introduction of the 4f system allows access to the microscope aperture and field diaphragms. In this configuration, the DMD is conjugated to the field diaphragm (FD) of the system to ensure the patterns projection onto the sample plane. In addition, a focus tunable lens (FTL) is located in the aperture plane of the 4f system to fine-tune the projection of the patterns onto the sample plane^36^. A render of the final assembled module is shown in panel (b) of Fig. 4. The corresponding optical elements are referenced both with its schematic identifier as text to describe the component and with a circled number to ensure they are easily distinguished in the rendering visualization.

The setting up of the microscope starts from the assembly of the Projection module. The idea is to align each part of the 4f system by making sure that a proper collimated beams comes out in the reverse path. To ensure an afocal system, collimation is guarantee by the use of a shear interferometer moved step-by-step^37^. As well as in conventional imaging, having small defocuses in the system due to optical alignment jeopardizes the SPM performance. Different solutions have been proposed to solve the said defocusing problem^33^. Nevertheless, the system itself should be checked to ensure precise operation conditions.

**Fig. 5:**
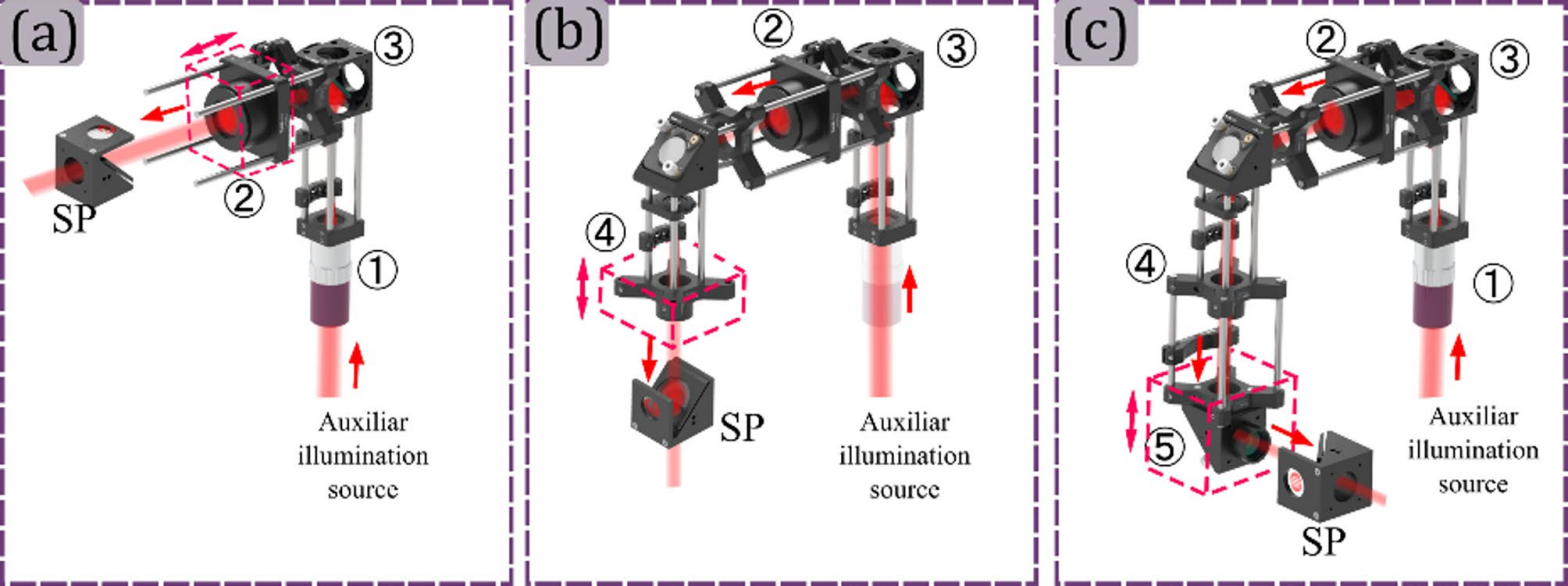
Projection Module assembly steps. Panels (a), (b) and (c) illustrate the setting up for guarantee the afocal configuration for the tube lens ②, lens ④ and lens ⑤, respectively. Panel (a) shows the afocal configuration between the microscope objective ① and the tube lens ②, panel (b) between the tube lens ② and lens ④, and panel (c) between lens ④ and lens ⑤. As is presented in the three panels, the correct position of the optical elements is defined using the shear plate (SP).

To do so, at the beginning of the M-Ⓐ assembly, a plane wave from an auxiliar coherent illumination source illuminates the microscope objective ① from the sample plane. Then the position of the tube lens ② is fixed by using a shear plate ensuring a proper alignment^37^. During this alignment, the beam splitter③ is placed between the microscope objective and the tube lens, as is shown in Fig. 5 panel (a). Then, the microscope objective ① is unscrewed, for illuminating the tube lens ② with a plane wave. Then, lens ④ position must be fixed by using the shear plate again, to ensure that a plane wave comes out of it, as is shown in panel (b) of Fig. 5. After that, the microscope objective ① is screwed in its position, and the lens ⑤ position is fixed by using the shear plate, guaranteeing the plane wave at the output of the system, as is shown in Fig. 5 panel (c).

### Reflected-light sensing module

**Fig. 6:**
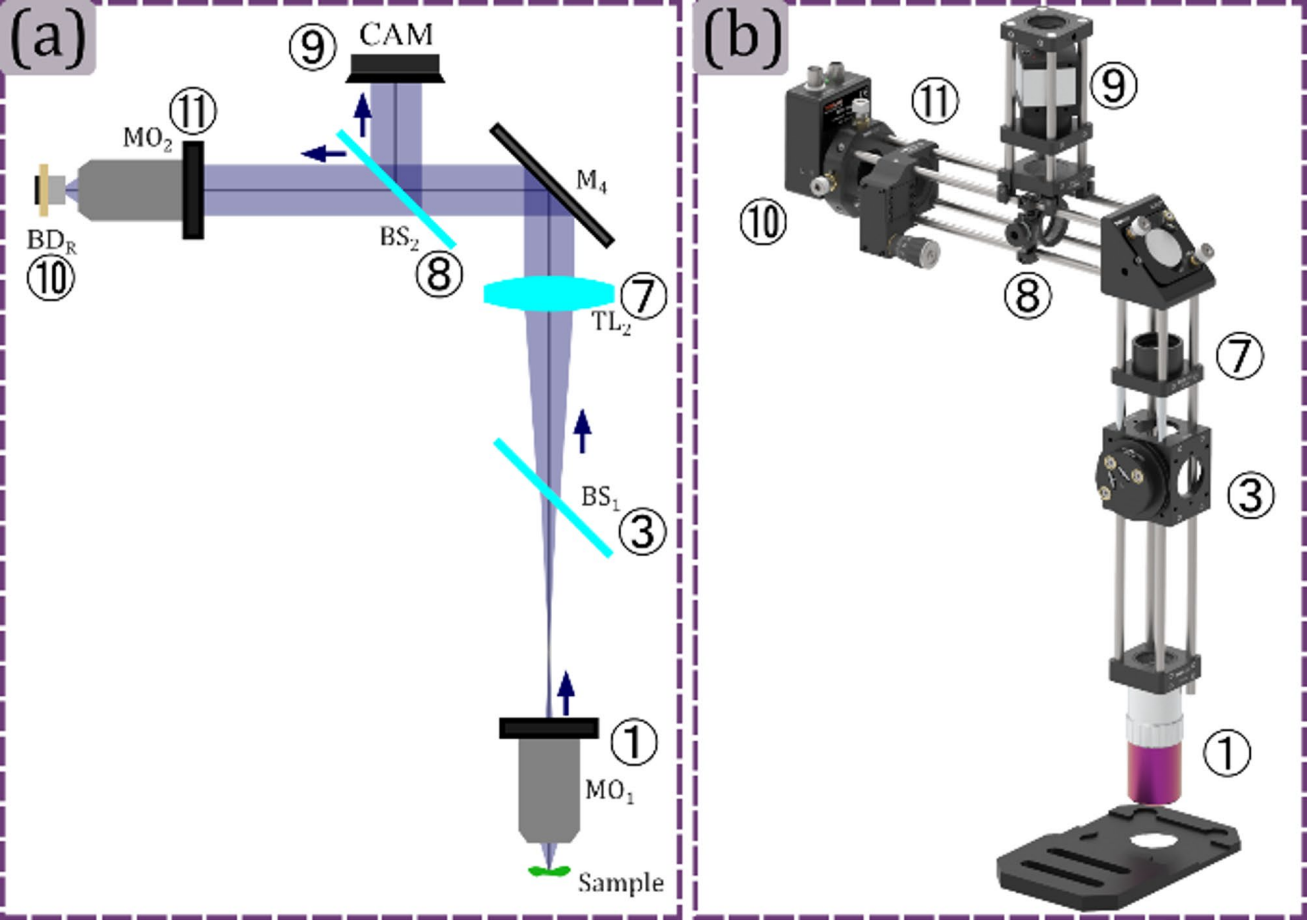
Reflected-light sensing module. In panel (a) Reflected-light sensing module schematic is shown. In panel (b) the rendered 3D view of the Reflected-light sensing module is shown, where each optical element is referenced using the corresponding number from panel (a). The following acronyms are used for the optical elements: Mi: Mirror, Li: Lens, TL**i**: Tube lens, BS**i**: Beam splitter, MO**i**: Microscope objective, BD**i**: Bucket detector.

In this module, the light reflected by the sample is acquired using a digital camera and a bucket detector, as is shown in panel (a) of Fig. 6. The first one is for checking the appropriate projection of the patterns on the sample plane, and the second is for the single-pixel detection. To ensure a correct magnification in the sample image over the camera plane, the lens ⑦ must have the same focal length as the tube lens ② and be placed after the beam splitter in a telecentric configuration with the microscope objective. After this lens, a 90:10 beam splitter ⑧ is used to divide the light into two paths with different intensity ratios. A control camera ⑨ has been included in the proposed setup for sample and pattern verification prior to SPM acquisition. The camera sensor is located at the focal length of the lens ⑦ in the 10% intensity light path of the beam splitter. This camera can also be used for data-fusion where a high spatial resolution sensor is typically processed alongside low-resolution spectral images acquired with SPM. The said measurements can be performed by changing one of the proposed detectors for a specialized one^34^. On the other hand, the bucket detector ⑩ is located in the 90% intensity light path to have most of the light for single pixel detector. In this path, a microscope objective ⑪ is used to focus the light onto the detector ⑩. The rendering of the assembled module is shown in Fig. 6b.

**Fig. 7:**
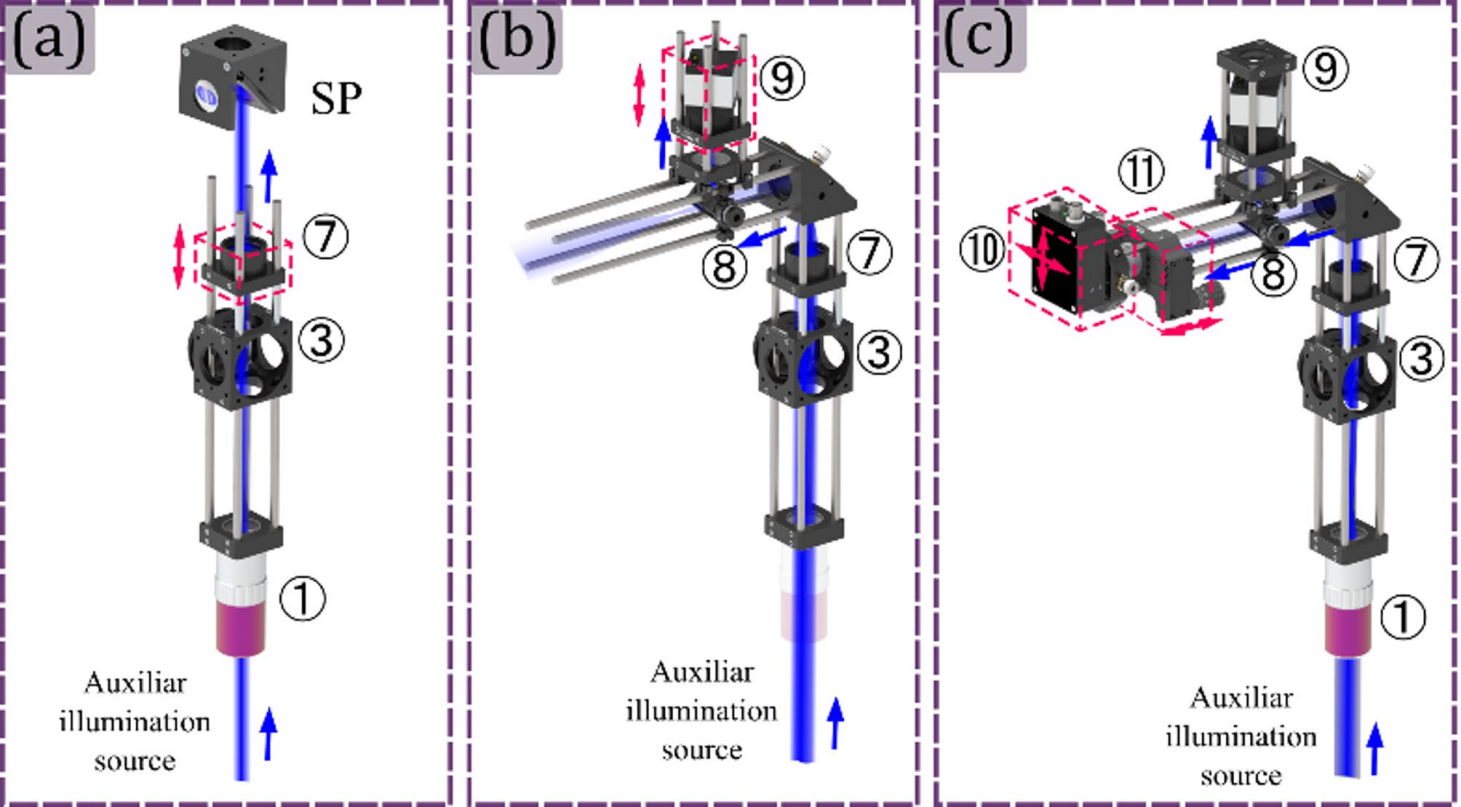
Setting up the reflection-light acquisition module. Panel (a) illustrates the configuration that ensures an afocal arrangement between the microscope objective ① and lens ⑦, utilizing the shear plate (SP). Panel (b) shows the placement of the control camera at the front focal plane of lens ⑦. Panel (c) shows the alignment of the photodiode ⑩, by the axial displacement of the microscope objective ⑪ and the lateral displacement of the photodiode ⑩. The following acronyms are used for the optical elements: SP: Shear plate.

This module can be assembled simultaneously as the Projection module. The position of the tube lens ⑦ is fixed by ensuring the telecentric configuration between this lens and the microscope objective ① using a shear plate, as is shown in Fig. 7 panel (a). Then, by removing the microscope objective ① and illuminating the lens ⑦ with a plane wave, the digital camera will be located at the focal length of the tube lens in the reflection module, as is shown in Fig. 7 panel (b); it is recommended to use a low-power laser for this and also to check laser induced damage threshold of the camera, and also use long exposure times so as not to compromise the device. Finally, the microscope objective ① is screwed in its position, and the light is focused on the single-pixel detector ⑩ by using a *xy* translation mount and a *z* translation mount for the microscope objective ⑪, as is shown in Fig. 7 panel (c).

### Transmitted-light sensing module

**Fig. 8:**
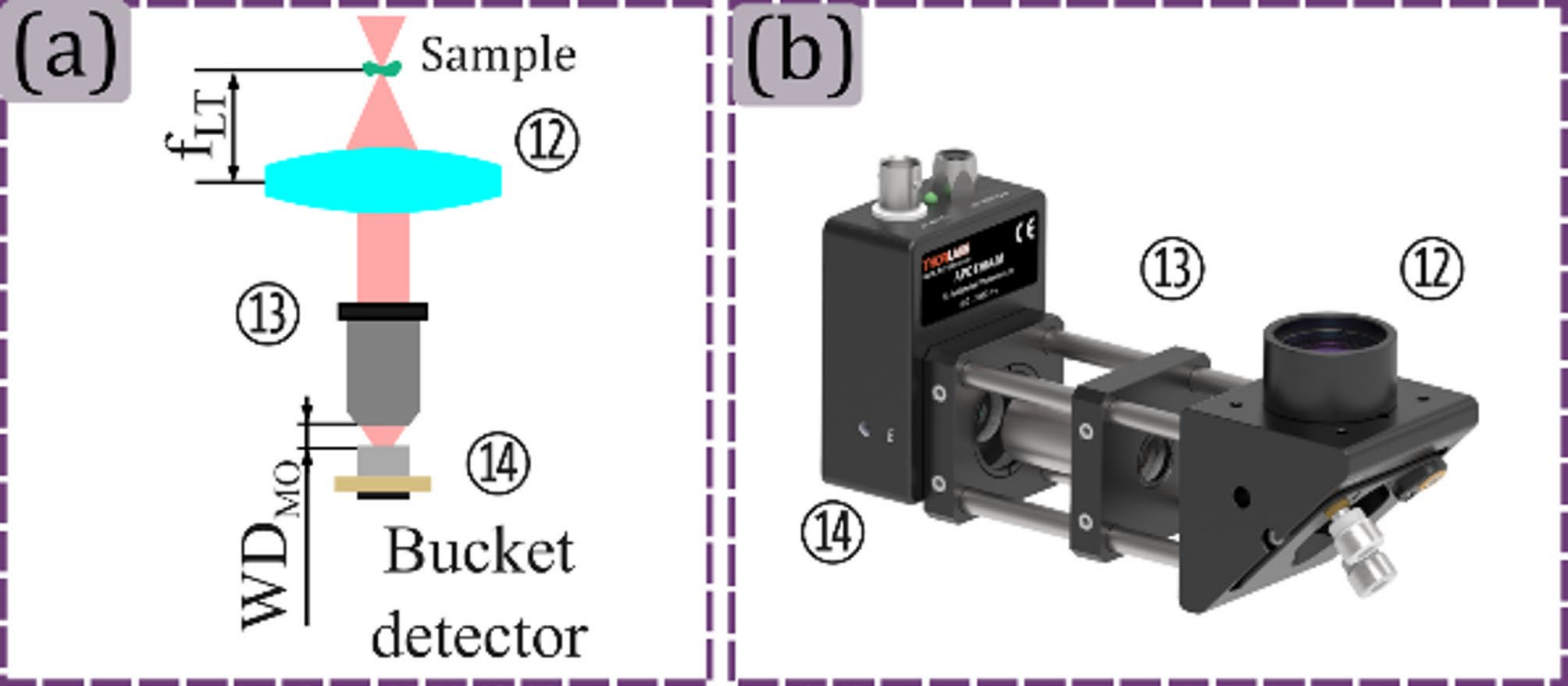
Transmitted-light sensing module. In panel (a) the transmitted-light sensing module schematic is shown. In panel (b) the rendered 3D view of the transmitted-light sensing module is shown, where each optical element is referenced using the corresponding number from panel (a). The following acronyms are used for the optical elements Li: Lens, Tli, MOi: Microscope objective, BDi: Bucket detector.

This module is simpler than the previous one, requiring only the single-pixel detection part. The light transmitted by the sample is somehow totally or partially collimated by a lens and then focused on the single-pixel detector using a microscope objective, as seen in Fig. 8a. The magnification of the microscope objective, similar to the reflected-light sensing module, must be selected according to the active area of the bucket detector to ensure that all the light is collected. In this module, the collimation lens ⑫ should be positioned for collecting all the light at the output of the microscope objective ⑬ and then focus it on the photodetector ⑭. The rendering of the assembled module is shown in Fig. 8b.

The bucket detectors included in both the Reflected-light and Transmitted-light sensing modules can be replaced by an optical fiber and then coupled to a spectrometer for multi- or hyperspectral imaging.

### Illumination coupling module

This module mainly defines the good coupling of the illumination in the single-pixel microscope by adequately integrating the DMD into the system. The use of DMD-based optical systems represents a rather challenging alignment process. This device consists of an array of independent micro-sized mirrors with controllable tilt angle states. The DMD has three states; when it is not energized, the tilt angle of the mirrors is 0°, which is called the flat state. On the other hand, when it is energized, the micromirrors flip between two tilt angles, $\:+\alpha\:$ for the ON state, and $\:-\alpha\:$ for the OFF state. Since $\:\alpha\:$ is typically 12°, the beam lighting the DMD at $\:24^\circ\:$ is reflected perpendicular to the DMD for the ON state, and is reflected at $\:48^\circ\:$ far from the optical axis for the OFF state^11,12^ .

**Fig. 9:**
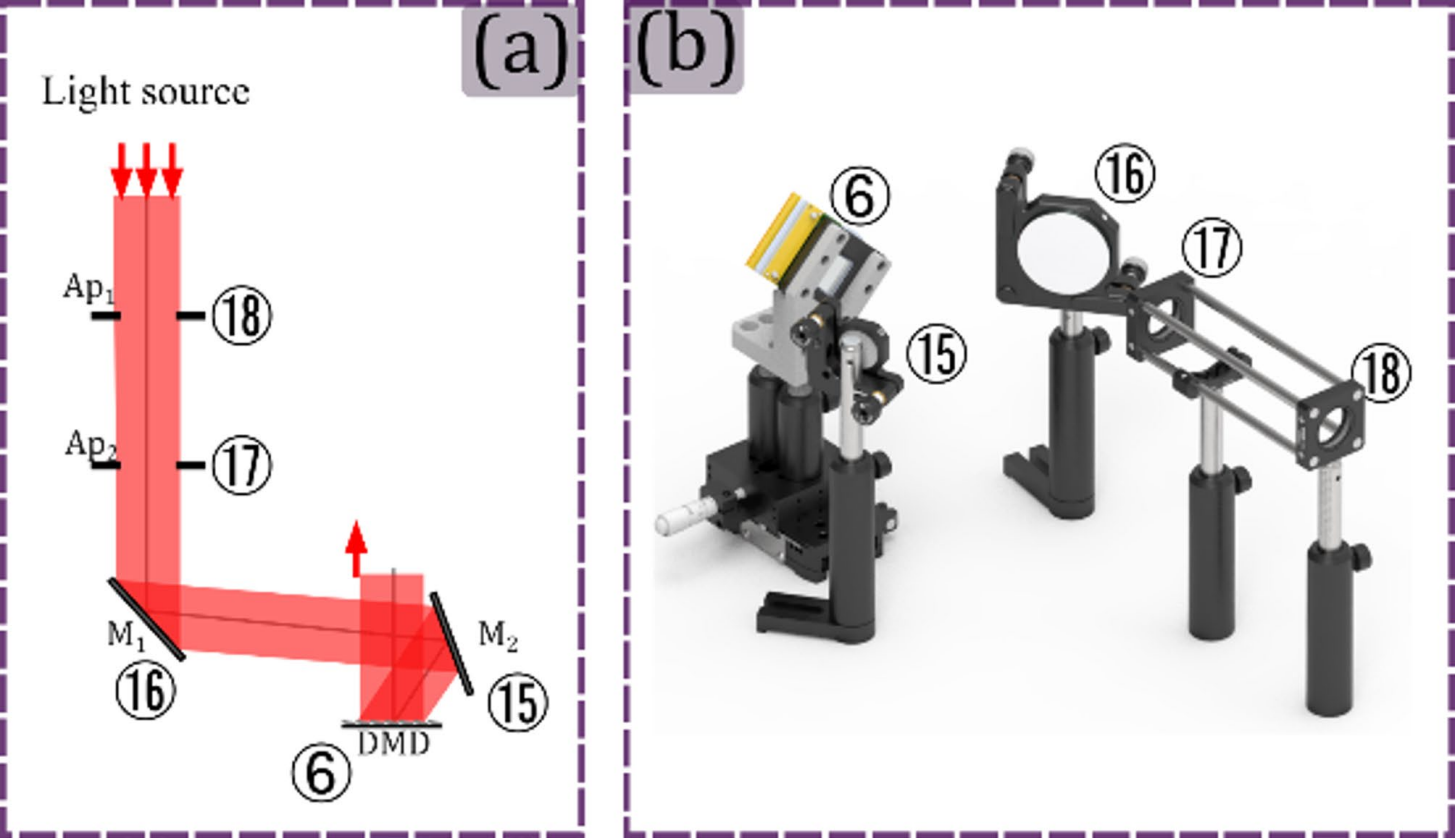
Illumination Coupling Module. In panel (a) the illumination coupling module schematic is shown. In panel (b) the rendered 3D view of the illumination coupling module is shown, where each optical element is referenced using the corresponding number from panel (a). The following acronyms are used for the optical elements Mi: Mirrors, Api: Diaphragm, DMD.

In practice, aligning the DMD to ensure that the illumination beam enters at the correct angle (typically 24°) is time-consuming and requires a high level of alignment expertise. The alignment difficulty also increases when multiple light sources are required. To overcome this, the proposed design includes an illumination coupling module consisting of two apertures and two mirrors, as is shown in panel (a) of Fig. 9. The light illumination passes through the two apertures and is then reflected by two mirrors, ensuring the light beam hits the DMD at the correct angle. Since the apertures define the optical axis, any light source aligned with the apertures will hit the DMD at the correct angle after being reflected by the mirrors. The render of this module is shown in Fig. 9 (b).

One could try to align the incident beam to the system by using the mirrors 15 and 16, nevertheless aligning the incident beam using only steering mirrors is often impractical and highly sensitive to errors. The DMD acts as a diffractive surface, and Gaussian beam propagation complicates angular alignment^38,39^. Small deviations in angle shift the reflected beam outside the system’s pupil, reducing image contrast and resolution. Previous approaches include introducing diffractive correction elements or adjusting mirror angle^39,40^. These strategies often add complexity and may degrade optical performance due to beam clipping or off-axis projection of the sampling patterns. Instead, we propose a robust alignment based on the Helmholtz reciprocity principle. An auxiliary collimated beam, launched through the imaging path, is used to locate the DMD normal. When the DMD is in its flat state, a properly aligned beam returns on its own axis. This condition confirms perpendicularity between the DMD and the optical axis. Once confirmed, the correct 24° incidence is achieved by setting the DMD in the ON state.

**Fig. 10:**
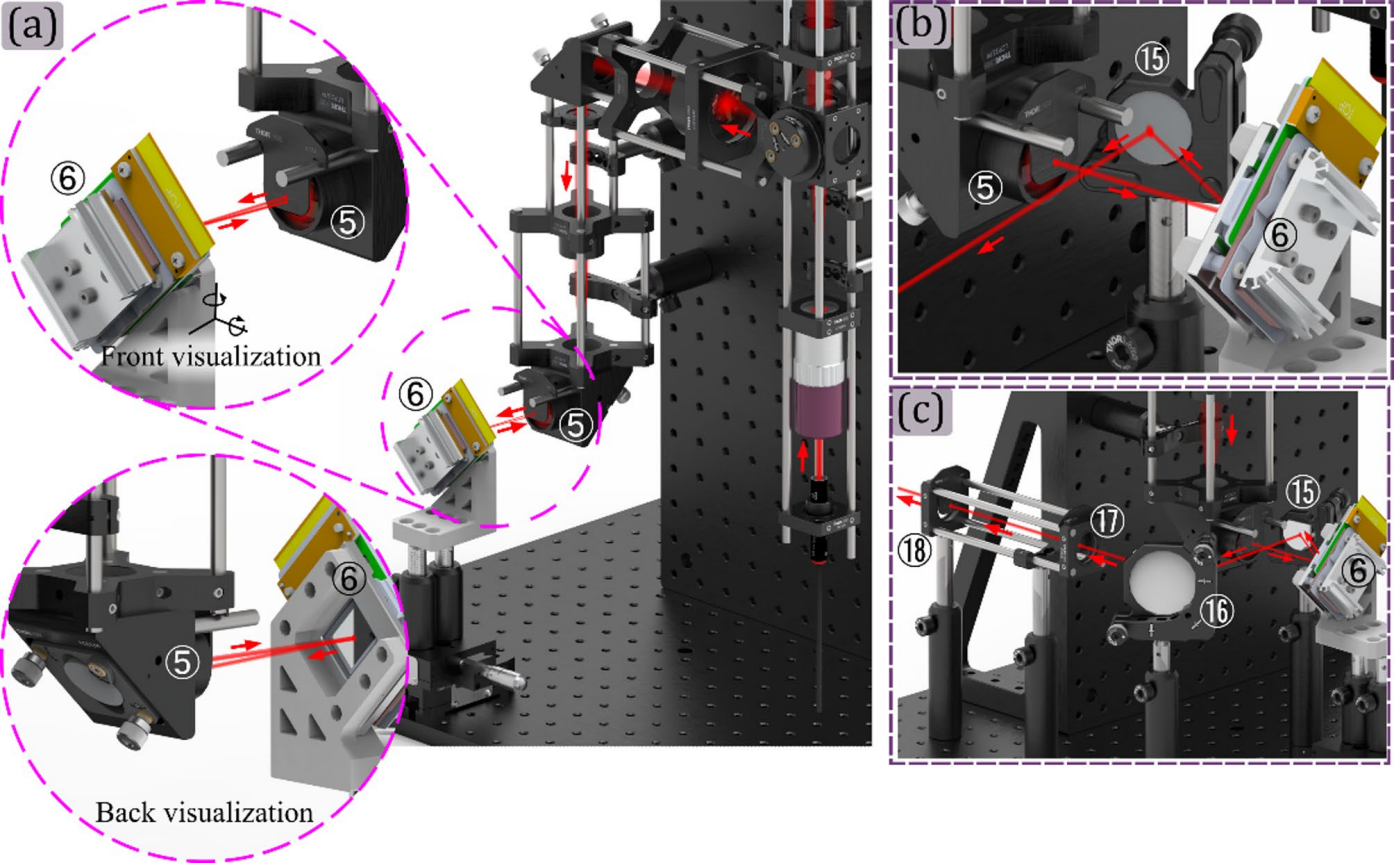
Setting up the illumination coupling module. Panel (a) illustrates the tuning of the angle of a beam that comes out from the microscope to ensure it is perpendicular to the optical axis established by the microscope and the DMD in a non-energized state. The front and back visualizations of the panel (a) also show that even a slight deviation from perpendicular alignment between the DMD and the optical axis can cause a tilt in the illumination. Panel (b) shows the light beam from the microscope hitting the mirror ⑮ when the DMD is set in the ON state. Finally, panel (c) shows the alignment of the optical axis in the center of the apertures ⑰ and ⑱ by tilting mirrors ⑮ and ⑯.

Mirrors are then placed to direct light along the known outgoing path. This method avoids the need for custom diffractive elements or complex angular calibration. It preserves beam collimation and ensures symmetric pattern projection. Figure 10 illustrates each alignment step in detail.

To align the module shown in Fig. 9, the imaging module must be set up and the DMD ⑥ must be positioned approximately at the front focal distance from the lens ⑤. After that, an auxiliary light source will be placed illuminating the microscope objective ① with a plane wave, following the optical axis of the projection module, and coming out of the system through the lens ⑤, as is shown in panel (a) of Fig. 10. Setting the DMD in the flat state, when a small beam coming from the lens ⑤ hits the DMD, it will be reflected perpendicularly along the same path only if the DMD is positioned perpendicular to the optical axis. Otherwise, a small tilt of the DMD will cause a tilt in the light beam, and the light would not return to the system in the same path, as shown in the zoom-in of the front and back visualizations of the light hitting the DMD in panel (a) of Fig. 10. Now, setting the ON state, the light incident perpendicular to the DMD is reflected at an angle of 24° along the illumination path, as shown in Fig. 10b.

At this angle, mirrors ⑮ and ⑯ are located in the setup respectively, as shown in Fig. 10c. Finally, the apertures ⑰ and ⑱ are positioned, and mirrors ⑮ and ⑯ are used to define the optical axis of the system at the center of the apertures. Since the microscope is aligned from the inside, the Helmholtz reciprocity theorem of light^35^ guarantees that any light source aligned with apertures ⑰ and ⑱, will also be aligned with the microscope system.

The illumination source is aligned with the microscope using the apertures ⑰ and ⑱. At this point, the transmitted-light sensing module can be coupled into the system, and the sample stage can be placed. The DMD must be energized and set on a completely white pattern to visualize a sample placed on the stage using the control camera ⑨. It is necessary to adjust the sample stage along the z-axis to achieve the proper focus of the sample. After that, a pattern is codified in the DMD ⑥. And the DMD is displaced across the optical axis to get the best focus visualization of the patterns into the control camera ⑨ image. Finally, the FTL ⊕ is positioned in the aperture plane of the 4f system.

## Experimental validations

**Fig. 11:**
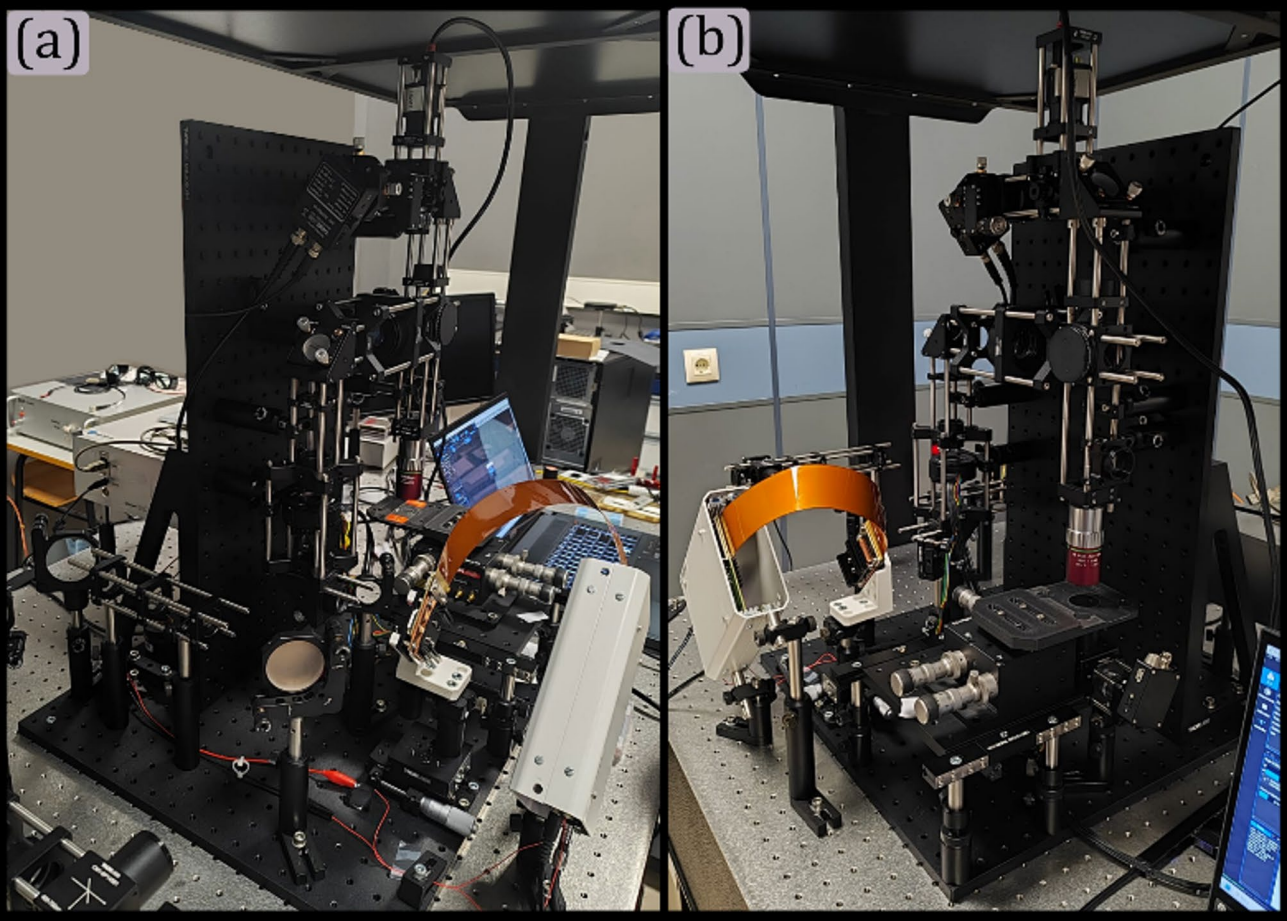
Panels (a) and (b) are pictures in different perspectives of the built single-pixel microscope.

A dual single-pixel microscope was built and aligned following the outlined procedures. The microscope was constructed using a Thorlabs 30 mm cage system. A Vialux V650L DMD, with a micromirror size of 10.8 μm per side served as the spatial light modulator (SLM) for pattern modulation. Two achromatic lenses with 75 mm focal lengths were used as lenses ④ and ⑤. A Thorlabs tube lens (TTL200-S8) and a 20X Mitutoyo microscope objective (Plan Apo NIR, 0.4 NA) were employed as key elements to demagnify the structured patterns onto the sample plane. The Reflected-light sensing module incorporated an avalanche photodiode (APD410A/M), while the Transmitted-light sensing module uses a silica photodiode (PDA36A2). A FTL ⊕ (Optotune EL-16-40-TC-20-C) was included to control pattern projection and correct defocusing for the HSPM. Additional details about the microscope components are provided in the following repository^41^. Panels (a) and (b) of Fig. 11 show pictures of the constructed microscope from different perspectives.

**Fig. 12:**
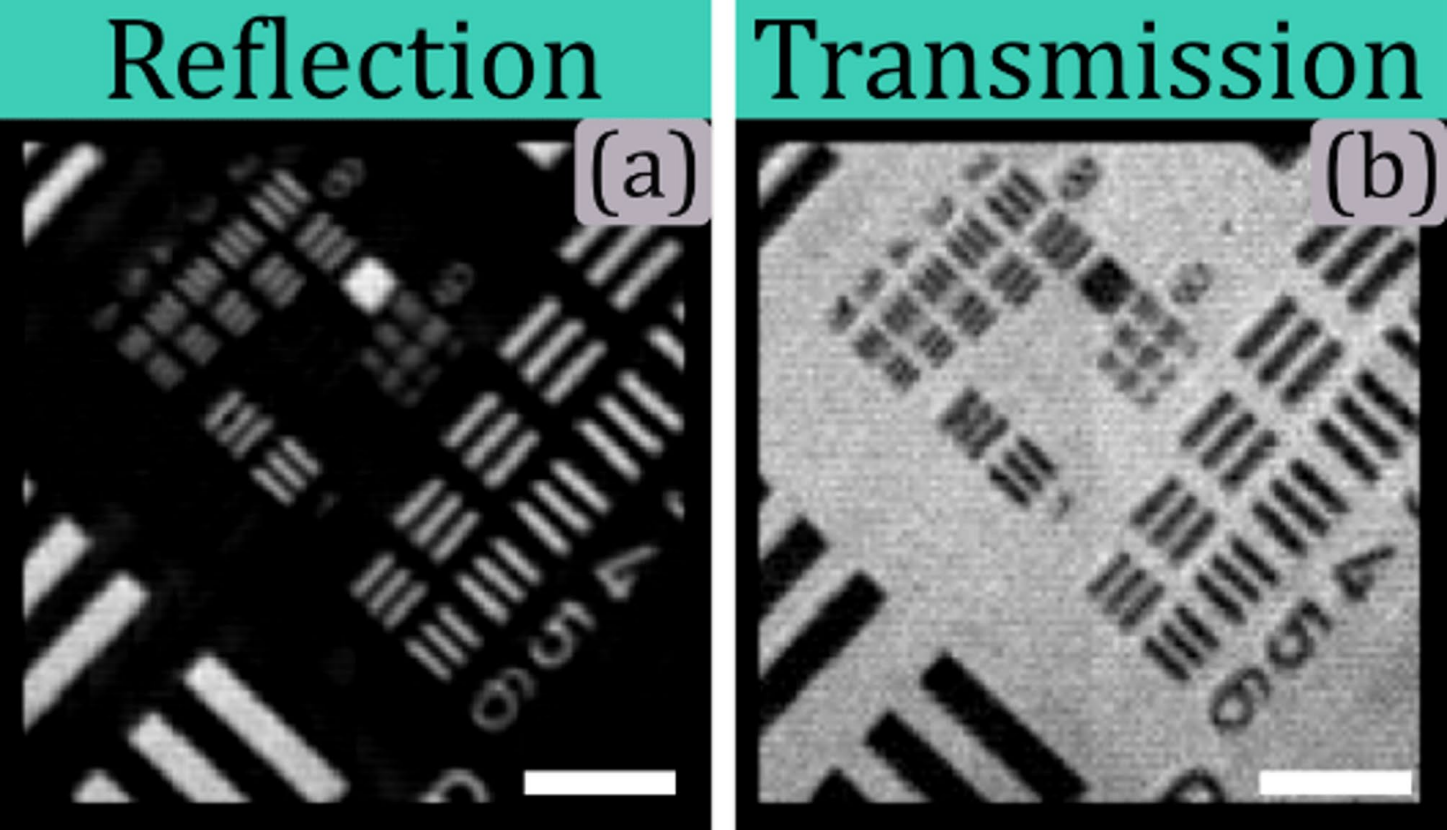
Resolution test chart imaged using the built dual HSPM. Panels (a) and (b) show the corresponding reflection and transmission images of a 1951-USAF test target using the proposed design. The scale bar corresponds to 30 microns.

Different tests with both biological and non-biological samples were conducted using the proposed SPM framework. Hadamard patterns of 128 × 128 with 2 × 2 micromirror binning were encoded on the DMD and projected onto the sample plane, yielding a theoretical resolution of 1.05 μm, as evaluated by Eq. (3). Autofocusing was achieved using the focus tunable lens (FTL) to maintain optimal focus^36^. The DMD speed was set to 10 kHz, while photodiode acquisition was performed using a NI-DAQ USB 6341, with each photodiode channel operating at 300 kilosamples per second. The acquisition time for the full 128 × 128 Hadamard basis was 3.3 s. The microscope’s performance was validated by imaging a positive reflective 1951-USAF test target, as shown in Fig. 12A 632 nm collimated diode was integrated into the microscope using the illumination coupling module described earlier in Fig. 9 the element 4 of group 8 is resolved, corresponding to an width of each element of ~ 1.38 μm. This performance surpasses recent single-pixel microscopy systems, which achieve up to 1.95 μm using higher NA and magnification microscope objectives^16^.

In Table 2 the information presented in Table 1^31^ regarding the imaging operational performance of different SPM is now complemented with the proposed design performance. In that way the proposed design can be directly compared with the previously mentioned developments. Reinforcing the proposed framework for design and implementation as a suitable way to standardize the HSPM implementation while reaching optimal configurations in terms of resolution.

Nevertheless, resolution is not only the key improvement. A significant advantage of the proposed microscope is also its dual acquisition mode. The reflected-light and transmitted-light sensing modules retrieve images simultaneously, as shown in Fig. 12, panels (a) and (b). Conventional microscopy typically requires two microscopes aligned for simultaneous reflection and transmission imaging. Unlike those solutions, in our design, the same scanning patterns are used, allowing light coupling via the transmission module for efficient dual-mode imaging and matching perfectly both acquired images. Additionally, the incorporation of ETL in our design allows the rapid change between different wavelengths without needed mechanical displacements to adjust the focal shift, just by implementing autofocus routines specifically designed for SPM.

**Table 2:** Proposed dual mode design operational parameters alongside the state-of-the-art solutions.

Previous reported	Imaging conditions	Resolution performance in terms of 1951 USAF target elements	Adaptable for reflection and transmission
Our proposed dual mode design	MO = 20x NA = 0.4	Group 8, element 4	Yes
^32^2013	NA	Group 4, element 5*	No
^2^2014	MO = 36x NA = 0.52	Group 7, element 3*	No
^25^2017	MO = 10x NA = 0.4	Group 7, element 3*	No
^13^2018	MO = 10x NA = 0.25	Group 7, element 4*	Yes
^31^2021	MO not applicable Magnification rate 0.1 Microscope with 300 mm and 30 mm focal length lenses	Group 6, element 6	No
^33^2023	MO = 10x NA = 0.25	Group 6, element 6	Yes
^16^2025	MO = 50x NA = 0.5	Group 9, element 1	No

*Computed based on the article presented results.

**Fig. 13:**
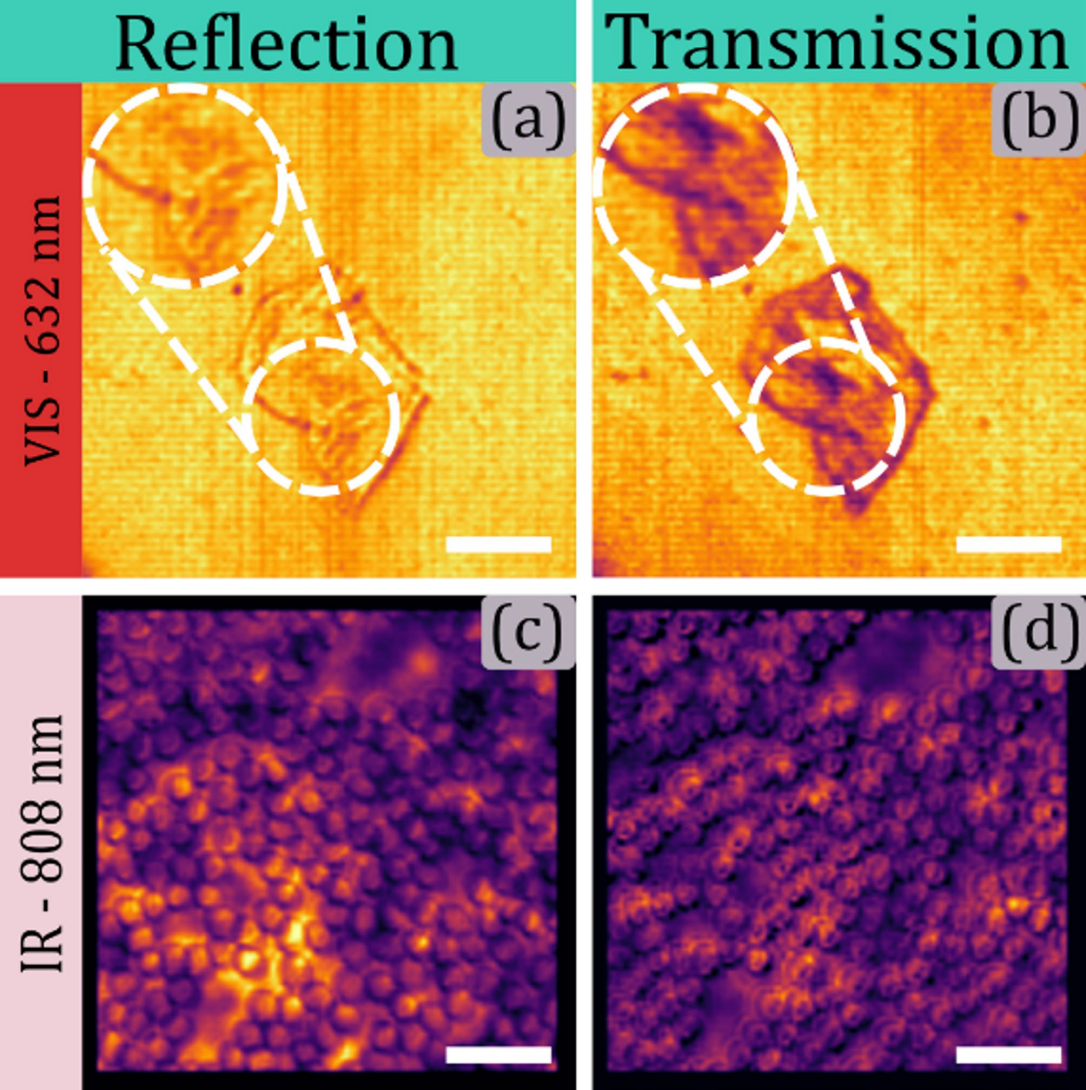
Dual imaging of biological samples. Panels (a) and (b) show Epithelial buccal cells in reflection and transmission configuration, respectively. Panels (c) and (d) show red blood cell smears in reflection and transmission configuration, respectively. The scale bar corresponds to 30 microns.

Epithelial buccal cells and red blood cells were imaged using the built HSPM, demonstrating its capabilities for biological imaging for the dual acquisition configuration. Epithelial buccal cells, which are semi-transparent, absorb visible light. The reflection image highlights cell borders, while the transmission image reveals nuclei and overall structure, as shown in Fig. 13, panels (a) and (b), respectively. Red blood cell smears, illustrated in Fig. 13, panels (c) and (d), challenging for coherent imaging due to light scattering in the visible range, were imaged effectively in the near-infrared, showcasing the system’s capability to analyze diverse samples.

**Fig. 14:**
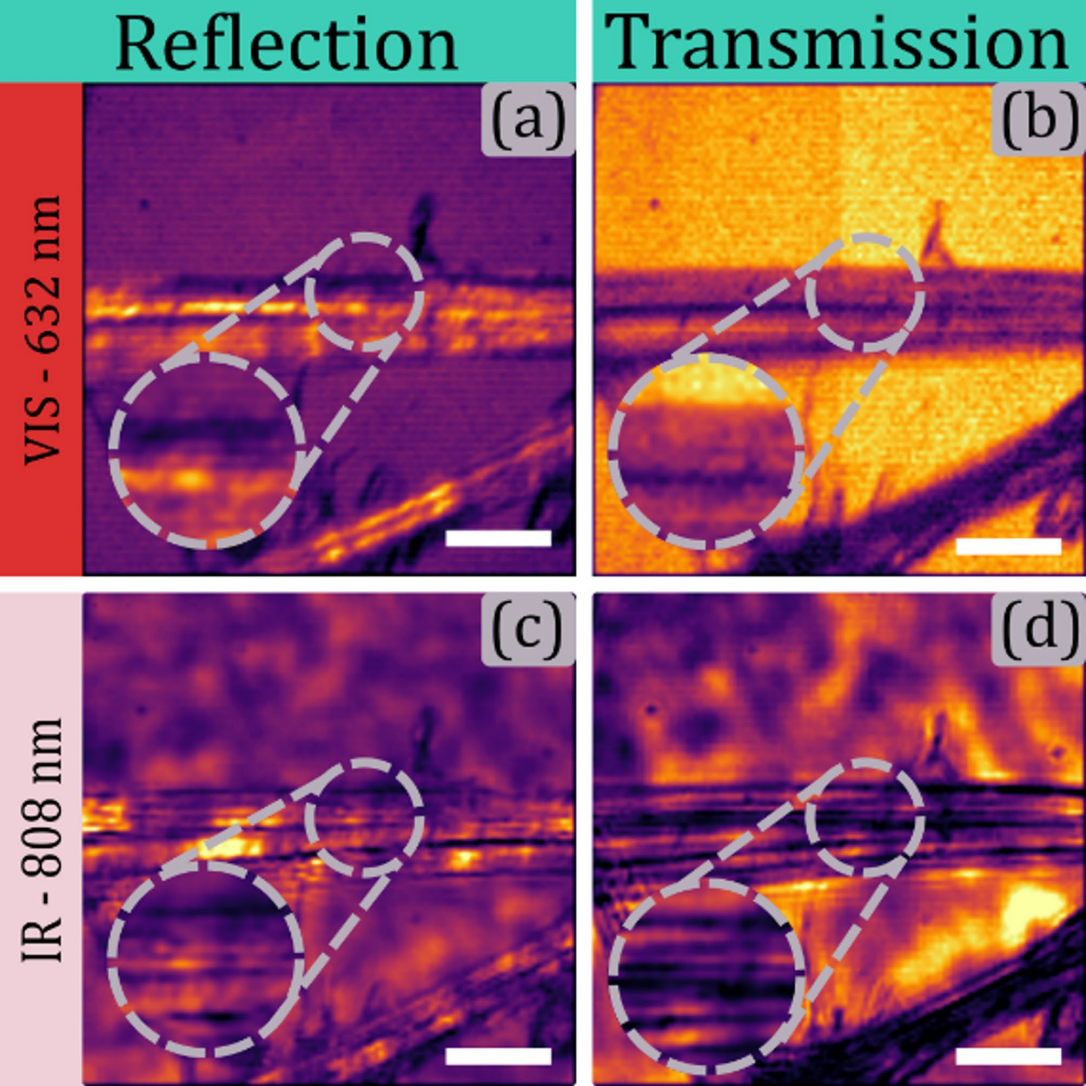
Dual imaging of cotton tissue for multiple wavelengths. Panels (a) and (b) show the reflection and transmission image for a 652 nm incoherent visible illumination, respectively. Panels (c) and (d) show the reflection and transmission image for an 808 nm coherent infrared illumination, respectively. The scale bar corresponds to 30 microns.

Finally, the system’s versatility extends to its interchangeable illumination sources. Figure 14 illustrates images of a cotton tissue obtained using both an incoherent 632 nm diode and a coherent 808 nm laser diode. While increasing the wavelength reduces resolution, it enables imaging of highly reflective samples in visible wavelengths by leveraging infrared light. Panels (a) and (b) show details captured using the 632 nm diode in reflection and transmission modes, respectively. Panels (c) and (d) present the same region imaged with the 808 nm laser diode, highlighting the different photon interactions at infrared wavelengths. Transmission images reveal hidden structural details not visible in the reflection mode.

## Conclusions

In this work, the design, assembly, and alignment procedures for a modular, active single-pixel-based microscope have been presented. The modalities of single-pixel microscopy (SPM) and its advantages have been discussed. Detailed, step-by-step instructions for constructing and aligning the system have been provided, with an emphasis on minimizing system aberrations and ensuring accurate projection of the structured light patterns. This work describes an open-source design and reproducible assembly procedures, contributing to the widespread adoption and dissemination of the SPM technique. Fundamental principles, such as the Helmholtz reciprocity theorem of light, have been employed to guarantee the correct alignment and positioning of the digital micromirror device (DMD) within the system. The procedure for aligning the DMD in active SPM systems has been described in detail.

The proposed design, incorporating a dual reflection and transmission configuration, offers a versatile, cost-effective solution for microscopy. Its modular and open-source nature makes it an accessible alternative to conventional microscopy systems. Performance evaluation has been conducted using a positive USAF resolution target, reaching a resolution of 1.55 μm, surpassing the capabilities of previously reported SPM configurations. Notably, biological validation experiments have been carried out using both visible and infrared illumination, demonstrating the system’s versatility across different modalities. The successful acquisition of coherent infrared images further highlights the system’s robustness and potential for its use in diverse microscopic applications in both biological and non-biological samples imaging.

This work addresses a gap in the current literature by providing a detailed methodology for optimizing single-pixel microscopes without the reliance on numerical or optical enhancement techniques, setting a new benchmark for SPM performance. The open-source, modular design presented here offers significant potential for advancing research in various fields, including biology, materials science, and environmental studies, by making high-performance imaging systems more accessible to a wider community of researchers. Future work may involve further optimization of the system for higher resolution or extending its capabilities for other imaging modalities, such as fluorescence lifetime imaging, to expand its potential applications.

## Data Availability

All the parts and assembly CAD files related to this work, including the list of materials, are available in the open access repository linked in reference^41^. For further details contact with the corresponding authors.
